# Draft Genome Sequence of Novel *Streptomyces* sp. Strain AJ-1, Isolated from a Leafcutter Ant in Uttarakhand, India

**DOI:** 10.1128/mra.00413-23

**Published:** 2023-06-20

**Authors:** Megha Choudhary, Bindu Naik, Vijay Kumar, Ankit Verma, Sanjay Gupta

**Affiliations:** a Himalayan School of Biosciences, Swami Rama Himalayan University, Dehradun, Uttarakhand, India; b Department of Food Science and Technology, Graphic Era Deemed to be University, Dehradun, Uttarakhand, India; DOE Joint Genome Institute

## Abstract

We present the draft genome sequence of *Streptomyces* sp. strain AJ-1, which was isolated from a leafcutter ant found in Uttarakhand, India. The genome assembly resulted in 43 contigs, with a combined length of 6,948,422 bp and a GC content of 73.5%. Through genome annotation, we identified 5,951 protein-coding genes and 67 tRNA genes.

## ANNOUNCEMENT

Ants harbor several beneficial microbiomes on their exoskeletons, including *Pseudonocardia and Streptomyces* species that provide a defense mechanism by producing antimicrobial substances (1–3). The present study was undertaken to isolate antimicrobial agent-producing actinobacteria associated with leafcutter ants in Uttarakhand, India. Ant samples were collected from the Dehradun district of Uttarakhand, India, on 20 December 2019 by using a net and were kept in sterile plastic containers until sacrificed for isolation of actinobacteria. Each ant was crushed in 500 μL of autoclaved Milli-Q water, the solution was plated on ISP-2 agar (4), and the plates were then incubated at 37°C for 5 to 7 days. The isolate AJ-1 was identified as *Streptomyces* sp. based on 16S rRNA gene analysis (GenBank accession number OQ256730), and the closest match was found to be Streptomyces daghestanicus NRRL B-5418 (GenBank accession number DQ442497). The actinobacterial culture was grown for 48 h at 30°C in 250-mL flasks containing 40 mL of nutrient broth (pH 7.2), at 180 rpm. The cell biomass was used for extraction of total genomic DNA (gDNA), following the method described by Kumar et al. (5). Briefly, cell biomass was redissolved in Tris-EDTA buffer containing lysozymes (20 mg/mL). After incubation at 37°C for 30 min, it was supplemented with 20 μL of proteinase K (1 mg/mL) and 10% SDS and was incubated at 50°C for 30 min. Phenol-chloroform was added, and the mixture was centrifuged at 10,000 rpm for 5 min at 4°C. The aqueous phase was taken and precipitated with 95% cold ethanol. The DNA was dissolved in DNA/RNA-free water and stored at 4°C until further use.

The gDNA concentration was determined with a Qubit 3.0 fluorometer and a Qubit double-stranded DNA (dsDNA) high-sensitivity (HS) assay kit (Thermo Fischer Scientific). A sequencing library was prepared using the NEBNext Ultra II DNA library preparation kit for Illumina (New England Biolabs). The amplified library was assessed for quantity and quality using a Bioanalyzer 2100 instrument (Agilent Technologies) with a HS DNA chip. Sequencing was conducted on the Illumina HiSeq 4000 platform, generating 6.2 million paired-end reads with an average read length of 151 bp in the 2 × 50-bp format. Trimmomatic v0.40 was utilized to perform quality trimming of the Fastq sequences, with default parameters. FastQC v0.11.2 was employed for quality assessment (https://www.bioinformatics.babraham.ac.uk/projects/fastqc). *De novo* assembly of the bacterial genome was carried out using Unicycler v0.5.0 with SPAdes as the *de novo* assembler (6). The resulting assembly consisted of 43 contigs, with a total length of 6,948,422 bp, an *N*_50_ value of 468,333 bp, a coverage depth of 270.06×, and a GC content of 73.5%. The draft genome annotation was performed using the NCBI Prokaryotic Genome Annotation Pipeline (PGAP) v6.1 (7). The annotation process identified 5,951 protein-coding sequences, 3 noncoding RNAs (ncRNAs), 67 tRNAs, 4 rRNAs (one 5S rRNA, one 16S rRNA, and two 23S rRNAs), and 62 pseudogenes. BAGEL4 v1.2 (8) was utilized to analyze gene clusters involved in secondary metabolite biosynthesis. The analysis revealed the presence of eight clusters, which were predicted to encode linocin M18, zoocin A, sactipeptides, thiopeptide, salinomycin, sactipeptides, lanthipeptide class II, and zoocin A.

### Data availability.

The draft genome sequence of *Streptomyces* sp. strain AJ-1 has been deposited in the NCBI database under BioProject accession number PRJNA967150, BioSample accession number SAMN34578207, assembly accession number GCA_029961205.1, and SRA accession number SRR24758175.
